# Anterior chamber paracentesis during intravitreal injections in observational trials: effectiveness and safety and effects

**DOI:** 10.1186/s40942-019-0157-z

**Published:** 2019-03-06

**Authors:** Sandeep Saxena, Timothy Y. Lai, Hideki Koizumi, Michel E. Farah, Daniela Ferrara, David Pelayes, Tomohito Sato, Carsten H. Meyer, Timothy Murray, for the International Pharmacokinetic Collaboration, Gabriel Andrade, Clement K. Chan, Sandeep Grover, Michel E. Farah, Sascha Fauser, Daniela Ferrara, Vincenco Ferrara, Roxane J. Hiller, Yusuke Ichiyama, Makoto Inoue, Marisa Hernandez Garfella, Saeed Karimi, Hideki Koizumi, Michael Koss, Timothy Y. Lai, Fung Liu, Carsten H. Meyer, Rajeev Muni, Timothy Murray, Sundaram Natarajan, Piergiorgio Neri, Masahito Ohji, Dong Ho Park, David Pelayes, Nelson A. Sabrosa, Christopher Riemann, Yoichi Sakurada, Tomohito Sato, Sandeep Saxena, Massaru Takeuchi, Sung Jae Yang, Han Zhang, Yingnan Zhang

**Affiliations:** 1 Department of Ophthalmology, King George’s Medical University, Lucknow, India; 2 Hong Kong Eye Hospital, Department of Ophthalmology and Visual Sciences, The Chinese University of Hong Kong, Hong Kong, Hong Kong; 3 Department of Ophthalmology, University of the Ryukyus, Okinawa, Japan; 4 Department of Ophthalmology, Federal University of Sao Paulo (UNIFESP), São Paulo, Brazil; 5 Department of Ophthalmology, Tufts University School of Medicine, Boston, Massachusetts USA; 6 Department of Ophthalmology, Buenos Aires University and Maimonides University, Buenos Aires, Argentina; 7 Department of Ophthalmology, National Defense Medical College, Tokorozawa, Japan; 8 Department of Ophthalmology, Smartlook, Aarau, Switzerland; 9 Bascom Palmer Eye Institute, University of Miami, Miami, FL USA

## Abstract

A paracentesis prior to an intravitreal injection is a very safe procedure and can prevent IOP-spikes after injections. As these spikes pose the risk of inducing glaucomatous changes particularly in patients with frequent injections and/or with a risk profile, a regular paracentesis prior to an injection may be considered and discussed with the patient.

## Intravitreal injections and acute increase of intra-ocular pressure

For decades, anterior chamber (AC) paracentesis has been a well-established, safe and cost-effective procedure to immediately reduce pathological elevation of intraocular pressure (IOP). The importance of AC paracentesis has regained attention with the widespread use of intravitreal injections after the approval of anti-vascular endothelial growth factor (VEGF) medications for the treatment of age-related macular degeneration (AMD) and retinal vascular diseases more than a decade ago [1]. It is evident that any injection of fluid into the vitreous cavity induces acute IOP increase, which is usually physiologically compensated within minutes to hours in the majority of patients [2]. However, in some patients, even the typically prescribed volume of 50 µl intra-vitreal anti-VEGF injection can cause acute vision loss and ocular pain secondary to acute IOP increase immediately after the injection [3]. When this happens, in many cases AC paracentesis is necessary to avoid permanent damage to the optic nerve. Persistent IOP increase may be present in some eyes and may cause acute angle closure attack [4].

There is some controversy about the clinical management of IOP increase in the post-intravitreal injection period [4]: while some reports showed a rapid IOP spike [5] and speculated that this might cause damage to the optic nerve, other authors believe this is negligible as IOP usually returns to normal within 15–30 min [6]. Nevertheless, there are patients with considerable IOP spikes of more than 80 mmHg post intravitreal injection that might be asymptomatic and therefore undetected after the injection or at the next day clinical check-up [7], which might result in serious and irreversible damage to the optic nerve and should have been treated immediately. One study indicated AC paracentesis in 33% (n = 87) out of 230 intravitreal injections and advocates on the benefits of such procedure [8]. All patients should be considered for AC paracentesis in the management of post-injection IOP spike regardless of injection volume, previous diagnosis of ocular hypertension or ocular globe size.

## Chronic intravitreal therapy and potential long-term side effects

Treatment algorithms for AMD with anti-VEGF injections have changed considerably over the past decade. “Real world” data showed that patients often received less than 5 injections per year [9] with suboptimal outcomes due to under-treatment. While treatment algorithms such as “*pro re nata*” or “treat and extend” aim to reduce the burden of monthly anti-VEGF injections, optimal outcomes such as those observed in clinical trials can only be achieved with more frequent treatments. At the other hand, long-term follow up in successfully treated neovascular AMD cases shows other causes of visual decline, such as geographic atrophy [10] or optic nerve atrophy [11]. In one study, Pershing et al. [12] observed that 4 years after anti-VEGF therapy, 81% of treated eyes developed unilateral glaucoma requiring IOP-lowering medication. Eyes treated with intravitreal injections showed significantly loss in the retinal ganglion cell layer (RGCL) compared to the untreated fellow eye over a period of 2–4 years [13, 14].

These can be found regardless of age and disease: the same effects of RNFL decrease were found in older patients treated with intravitreal injections for AMD and in younger patients treated for diabetic macular edema [15]. There seems to be no difference in risk of RNFL damage between intravitreal triamcinolone or anti-VEGF drugs, suggesting that increased IOP and not a drug-specific mechanism may be the underlying cause [16].

## Therapy options and paracentesis risk assessment

European retina specialists, through the EURETINA expert’s consensus recommendations of 2018, reported that 89% of patients submitted to intravitreal injections experienced IOP increase above 30 mmHg 5 s after the procedure, and in approximately one third of these patients the IOP remained high during the first 5 min [17]. A pre-treatment AC paracentesis or tap can reduce the impact of transient IOP elevation and was lately conformed in a 2018 literature searches of the PubMed and Cochrane databases by the American Academy of Ophthalmology [18]. Some authors believe that frequent IOP spikes after intravitreal injections can lead to unilateral glaucoma of difficult clinical management, which then might require a surgical procedure to prevent further progression. Meyer et al. postulated that the problem might be related to injection volumes greater than 50 µl due to an improper calibration and preparation of intravitreal syringes. These authors measured a range of injection volumes in a clinical routine setting: from 0.24 to 0.65 μl observed for an intended 50 μl injection volume [19].

The IOP increase associated with intravitreal injection has also been explained by a biomechanical model, in which an injection volume of 100 µl resulted in IOP increase up to 40.6 mmHg. Eyes with shorter axis length showed greater response in one study [20]. Injection volumes greater than 50 µl were previously thought to be more commonly associated with IOP spike, as shown after the administration of 90 µl pegaptanib (Macugen, Pfitzer) inducing IOP spike greater than 50 mmHg in 45% of patients, which motivated some physicians to consider prophylactic AC paracentesis [21]. However, more recent studies did not find a clear association between intravitreal injection of 100 µl and increased risk of clinically significant IOP spike, in comparison to the previous literature.

Some authors have recommended prophylactic paracentesis prior to intravitreal bevacizumab injections based on two arguments: first, the immediate IOP spike may damage the retinal microcirculation, potentially aggravating an already impaired blood-retinal barrier in diabetic or venous occlusive eye disease; second, AC paracentesis would prevent drug reflux, resulting in more medication entering the vitreous cavity. The pre-injection paracentesis can prevent the reflux ensuring the complete dose in the vitreous cavity. The incidence of complications is low when caution is maintained [22, 23].

## Risks of AC paracentesis

The incidence of complications related to AC paracentesis may be low when caution is exercised [24, 25]. Numerous reports evaluated the risk of AC paracentesis in vast experience for more than 20 years [24–27]. Potential complications could include pain, traumatic injuries of the iris, occurrence of AC fibrin, hyphema, severe inflammation, infection or persistent leakage with hypotension or endophthalmitis. Decades before the introduction of frequent VEGF-injections, Helbig et al. [28] reported a single case of bacterial endophthalmitis after the AC-paracentesis in an eye with a central arterial occlusion. Although disinfection and paracentesis were performed in an operating room, no eyelid speculum or drape was mentioned in the article. This preventive measure is today an essential part of the guidelines for intravitreal injections set up by the national and international ophthalmological societies. Helbig stated when questioned on personal request, that he assumed the use a lid speculum. However, he has observed no further infections ever since after any paracentesis during an intravitreal injection period for the last 30 years (personal communication by *Prof. Horst Helbig*, August 2018). Of course, this procedure remains not completely free of possible injuries: Meyer et al. [29] reported as complications only two posterior lens and one anterior lens injuries after the AC paracentesis in 32,318 cases. This favorable risk profile is further confirmed by the authors who did not experience any injury or infection in thousands of patients with AC-samples.

## Effectiveness of protective effect of paracentesis in intravitreal injections

Several studies demonstrated the effectiveness of prophylactic AC paracentesis in the prevention of immediate or long-term IOP increase associated with intravitreal injections [30–46]. Ichiyami et al. [41] studied 111 patients who received AC paracentesis prior to each intravitreal injection over a period of 12 months. No IOP increase was observed, defined as a change of more than 6 mmHg or IOP > 22 mmHg. Bach et al. demonstrated the effectiveness of paracentesis following intravitreal drug injections into maintain the physiologic ocular perfusion in 1681 cases. A median (SD) of 210 µl (40 µl) of aqueous was removed during each paracentesis and there were no reported incidences of endophthalmitis, capsular rupture, wound leak, AC collapse or any other negative outcome [47]. Soheilian et al. [48] monitored IOP values in 90 eyes 2 min, 30 min, 24 h, and 3 months post-injection. In group A, only the intravitreal injection was administered, in group B, an intravitreal injection was combined with AC puncture. In Group A, the IOP increase was 26.4 mmHg, 6.5 mmHg, 0.2 mmHg, and 0.5 mmHg in each time point respectively. In Group B, the relative IOP increase was − 1.3 mmHg, − 3.2 mmHg, 3.1 mmHg, and − 1.8 mmHg. The RNFL thickness was also measured in both groups at the same time points. The RNFL baseline values did not differ significantly (85.3 μm and 85.6 μm in groups A and B, respectively). However, after 3 months the RNFL loss in group A was − 2.0 μm and in group B only 0.2 μm. Enders et al. [49] evaluated 76 AMD patients without glaucoma treated by intravitreal injection with and without AC paracentesis. Again, there was a significant difference in RNFL decrease in treated eyes with and without paracentesis. The RNFL remained unaffected in patients with unilateral AC paracentesis after intravitreal injection compared to the untreated fellow eye.

## Take home message

AC paracentesis is a safe and effective option to manage acute IOP increase secondary to intravitreal pharmacotherapy. In addition, prophylactic AC paracentesis immediately before intravitreal injection is a safe procedure and prevent IOP spikes associated with this common treatment modality. As IOP spikes pose the risk of inducing glaucomatous changes in the optic nerve head, particularly in patients receiving frequent intravitreal injections, the option of prophylactic AC paracentesis and its risk/benefit profile should be considered and discussed with the patient.
